# Physiological and Biochemical Responses of Commercial Strawberry Cultivars under Optimal and Drought Stress Conditions

**DOI:** 10.3390/plants12030496

**Published:** 2023-01-21

**Authors:** Seyed Morteza Zahedi, Marjan Sadat Hosseini, Narjes Fahadi Hoveizeh, Saeid Kadkhodaei, Marek Vaculík

**Affiliations:** 1 Department of Horticultural Science, Faculty of Agriculture, University of Maragheh, Maragheh 83111-55181, Iran; 2 Department of Agriculture, Goldaru Pharmaceutical Company, Isfahan 81791-35111, Iran; 3 Department of Horticultural Science, College of Agriculture, Shahid Chamran University of Ahwaz, Ahwaz 61357-83151, Iran; 4 Agricultural Biotechnology Research Institute of Iran (ABRII), Isfahan Branch, Agricultural Research, Education and Extension Organization (AREEO), Isfahan 84156-83111, Iran; 5 Department of Plant Physiology, Faculty of Natural Sciences, Comenius University in Bratislava, Mlynská Dolina B2, Ilkovičova 6, 842 15 Bratislava, Slovakia; 6 Institute of Botany, Plant Science and Biodiversity Centre, Slovak Academy of Sciences, Dubravska Cesta 14, 845 23 Bratislava, Slovakia

**Keywords:** adaptation, antioxidants, camarosa, field capacity, proline, stress tolerance

## Abstract

Improving the extent of adaptation and the choice of the most tolerant cultivar is the first step to mitigating the adverse effects of limited water, especially in susceptible plants such as strawberries. To address this issue, two commercial strawberry cultivars (Camarosa and Gaviota) were compared when irrigated to match 100, 75, 50, and 25% field capacity (FC) to simulate the control, slight, moderate, and severe drought stress conditions, respectively. Drought stress induced the reduction of total chlorophyll, carotenoid, relative water content, and phenolic content significantly, whereas the activity of antioxidant enzymes, electrolyte leakage, osmolyte accumulation, and oxidative markers upsurged progressively in drought severity-dependent behavior. Gaviota produced more proline, hydrogen peroxide as a marker of membrane lipid peroxidation and disposed of by higher electrolyte leakage, significantly. On the other hand, Camarosa having higher soluble carbohydrates as well as enzymatic and non-enzymatic antioxidants could be considered a drought-tolerant cultivar. Genotypic variation between these cultivars could be used in breeding projects to promote drought-tolerant strawberries in the future.

## 1. Introduction

Strawberry (*Fragaria ananassa* Duch.) is an attractive small fruit with global regard due to its appreciable taste accompanied by the presence of various biological compounds. During a chemical analysis of strawberry fruit, molecules including antioxidants, phenolics, carotenoids, and ascorbic acid as well as minerals, vitamins, and sugars are all found at high concentrations [1]. In 2020, the production of strawberries in China amounted to approximately 3.3 million tons. China, the United States of America, and Mexico produce together more than 50% of the world’s total strawberries globally. Other top strawberry producing countries in the world include Egypt, Mexico, Russia, Japan, Republic of Korea, Poland, and Germany. The production of strawberries as an economically valuable product has doubled during the last decade. Today, 0.53% of global strawberry production is accomplished in Iran [2].

Drought stress as a serious environmental threat jeopardizes the yield of crop plants in Iran economically [3]. Drought is a major prevalent environmental stress limiting strawberry productivity and affecting its morphology as well as enzymatic and physiological activities [4]. Strawberry due to their shallow root system, large leaf area, and juicy texture, is significantly damaged from water deficiency, thereby reducing their biomass and crop yield [5]. It is noticed that plants greatly suffer from mineral shortcomings due to the unavailability of enough water in the soil and lower mobilization of minerals in the plants [6]. The decrease of growth parameters, relative water content (RWC), photosynthetic pigments, and photosynthesis itself occur due to stomatal closing and limitation of CO_2_ diffusion into leaves under drought stress. Therefore, less growth as well as oxidative stress are inevitable. Water deficiency leads to chlorophyll (Chl) degradation and direct suppression of photosynthesis which in turn affects plant development adversely by declining cell division, enlargement, and differentiation [7]. In stressful conditions, the photosynthetic efficiency of photosystem ΙΙ is substantially damaged due to the excessive release of reactive oxygen species (ROS) [8]. Water deficiency triggers the imbalance between ROS scavenging and their generation. This oxidative stress due to excessive concentration of ROS disrupts the photosynthetic apparatus containing chloroplast structures and reaction centers [9]. In reviewing data of recent years, with regard to the decrease in rainfall, dry and semi-arid conditions overcome in most parts of Iran and other regions of the world, troubles have arisen in the fruit growing industry including declining in the fruits quality as well as quantity. Therefore, this gradual water shortcoming necessitates the determination of drought-tolerant cultivars among existing ones [10]. Previously, the effect of drought stress on three strawberry cultivars was evaluated to detect the most tolerant responsive one [11]. Meanwhile, Ibrahim et al. [12] already reported that there was a significant superiority of California cultivar among three genotypes (Earlibrite, California ad Sweet Charlie) to encounter a limited level of irrigation water. In another experiment all the studied strawberry cultivars were impacted markedly by drought, nevertheless, two cultivars were less affected than the others [10]. Thoughts had been made to prove this hypothesis that the highest drought tolerance among various cultivars depends on genetic compatibilities. 

Plants have developed mechanisms that include morphological, physiological, and molecular responses which can differ between genotypes and drought severity [13]. More tolerant cultivars develop more morphological, biochemical, and physiological mechanisms than other cultivars under stressful conditions. The limitation in leaf area probably plays a role as the first defense in mediating the response of plants to drought. Tolerant cultivars maintained leaf water potential at a higher level compared to sensitive cultivars [14]. Furthermore, tolerant plants accumulate soluble compatible substances including proline and soluble sugars without hampering enzyme activities which, thereby, decreases the water potential [15]. Thus, tolerant plants modulate the adverse effects of drought stress through photosynthetic adjustment, osmolytes accumulation, and activities of genes that encode antioxidant enzymes [16]. Under drought stress, plants hamper transpiration phenomenon through different mechanisms such as closing the stomata, and developing stomatal resistance parallel with decreasing stomatal conductivity [17].

Regarding the high nutritional and economic value of strawberries and the variation in response of different cultivars to environmental stresses such as drought, an assessment of the mechanisms of this plant to cope with drought stress to select drought-tolerant cultivars is necessary. Therefore, the main objective of this research was to evaluate the response of two commercial strawberry cultivars to three levels of drought conditions to find out more tolerant one.

## 2. Results

### 2.1. Photosynthetic Pigments, Electrolyte Leakage (EL), and Relative Water Content (RWC)

Severe drought exhibited a remarkable reduction of 71, 38, and 32% in total Chl, carotenoids, and RWC in Camarosa and about 17, 53, and 33% in Gaviota versus normal conditions. By contrast, among different drought levels, severe drought caused a significant elevation of electrolyte leakage (EL) by 127 and 183% in Camarosa and Gaviota over normal growth conditions, respectively.

Without drought stress, Camarosa elucidated higher total Chl compared with Gaviota (23%), but the Gaviota plants exposed to severe drought, displayed more amount of total Chl (121%) comparing Camarosa plants in the same conditions. There was no significant difference among various drought stress levels between Camarosa and Gaviota in terms of carotenoid content. Furthermore, Camarosa strawberry plants showed more EL in control conditions (10%), whereas, in severe stressful conditions, more EL was observed in the Gaviota plants (5%). In slight and moderate drought, the RWC in Camarosa was higher about 5% and 8%, respectively, compared to Gaviota (Table 1).

### 2.2. Antioxidant Enzymes Activity

Severe drought stress caused an increasing tendency in the contents of peroxidase (POD), catalase (CAT), superoxide dismutase (SOD), and ascorbate peroxidase (APX) by 137%, 220%, 47%, and 124% in Camarosa and 121%, 300%, 38%, and 93% in Gaviota over non-stressed strawberry plants.

Values are means ± SD (*n* = 3). Different letters within the same column indicate significant differences at *p* < 0.01 among the treatments, using Duncan’s multiple range test.

Gaviota cultivar exhibited more activity of POD by 15% and 7% in control and severe drought stress, respectively, as compared with that of Camarosa plants. Conversely, Camarosa showed significantly higher activity of CAT in different levels of drought (*p* < 0.01). Both moderate and severe drought stress led to higher SOD activity in Camarosa by 9% but Camarosa only manifested higher APX activity in severe stress (10%) relative to its respective amount observed in the Gaviota cultivar (Table 2).

### 2.3. Oxidative Markers and Osmolytes

H_2_O_2_ and malondialdehyde (MDA) as oxidative markers profoundly indicated an increasing trend with intensifying drought stress by 36% and 28% in Camarosa and 94% and 59% in Gaviota, respectively, as compared with control. Camarosa plants accumulated higher osmolytes of proline and total soluble carbohydrates by 71% and 44%, respectively, in severe drought stress conditions over the no-stress condition. On the other hand, Gaviota accumulated higher proline (127%) and less total soluble carbohydrate (24%) in severe drought as compared with non-drought conditions.

Under moderate and severe drought stress, the Gaviota cultivar displayed remarkably higher H_2_O_2_, but MDA was higher in Camarosa in all drought stress levels under either non-drought or drought conditions in comparison with Gaviota. Proline as an important osmolyte, accumulated in Gaviota cultivar in higher amounts over Camarosa in moderate and severe drought stress by 37% and 40%, respectively. However, there was no significant difference between cultivars in this regard comparing control and slight drought stress condition. Eventually, Camarosa significantly (*p* < 0.01) accumulated higher total soluble carbohydrates than Gaviota in all drought stress levels (Table 3).

### 2.4. Phenolic Content and Antioxidant Activity (DPPH Assay)

Phenolic content, as well as DPPH (2,2-diphenyl-1-picryl-hydrazyl-hydrate), decreased in all drought stress levels including severe drought by 58% and 55% in Camarosa over the non-stressed condition. Gaviota displayed a 64% decrease in both phenolic content and DPPH as a result of severe drought stress over without stress.

**Figure 1:**
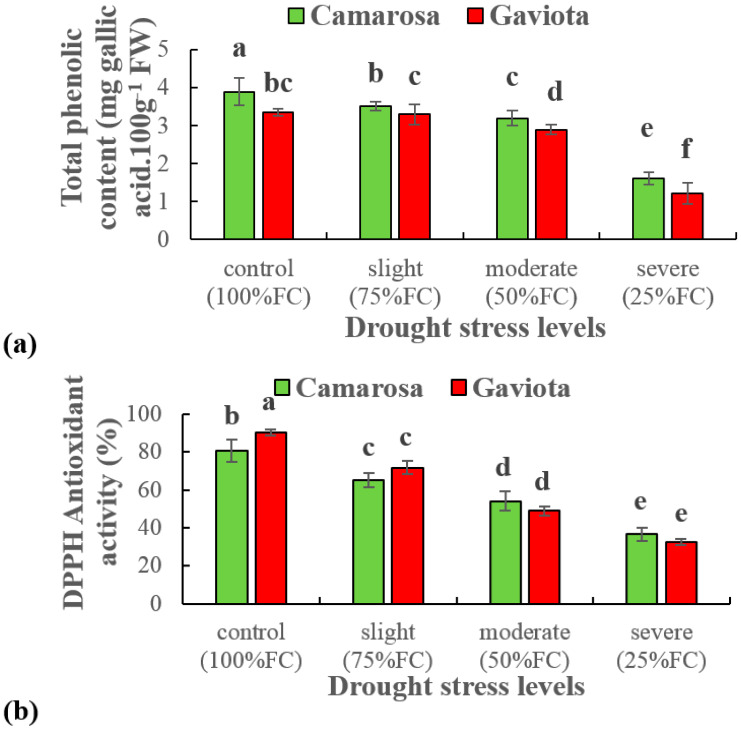
Effects of different drought stress levels on total phenolic content (**a**) and DPPH antioxidant activity (**b**) of two strawberry cultivars (Camarosa and Gaviota). Values are means ± SE (n = 3). Values with different letters are significantly different at *p <* 0.05 according to Duncan’s multiple range test.

All levels of drought stress resulted in higher biosynthesis and accumulation of phenolic content in Camarosa than in Gaviota. However, despite showing significantly (12%) higher DPPH by Gaviota in the control condition, Camarosa plants exposed to different levels of drought stress expressed significantly higher DPPH than the Gaviota cultivar (Figure 1).

### 2.5. Heat Map Analysis

**Figure 2:**
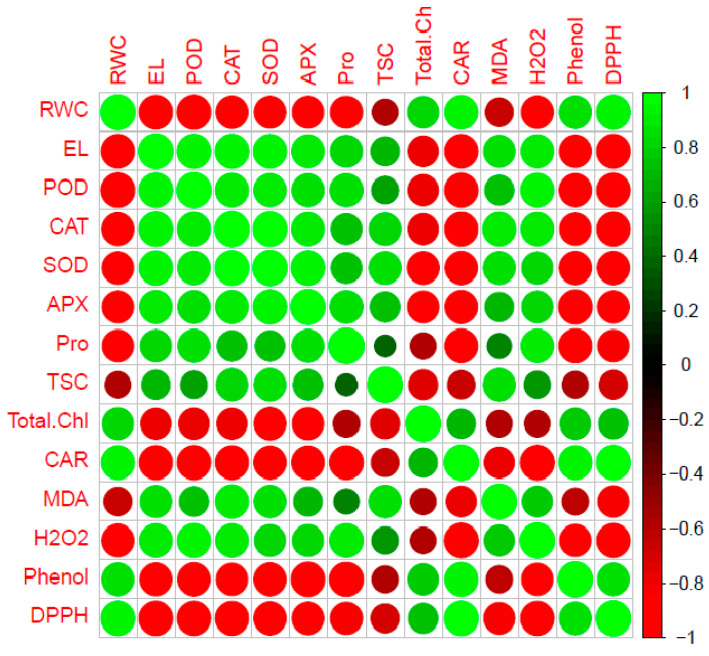
Heatmap of Pearson correlation coefficient of all evaluated traits in two strawberry cultivars (Camarosa and Gaviota) at four drought stress levels (100, 75, 50, and 25% FC). The right height axis exhibits positive and negative correlations presented in green and red colors, respectively, according to the color scale, RWC, relative water content; EL, electrolyte leakage; POD, peroxidase activity; CAT, catalase activity; SOD, superoxide dismutase activity; APX, ascorbate peroxidase activity; Pro, proline; TSC, total soluble carbohydrates; Total Chl, total chlorophyll; CAR, carotenoids; MDA, malondialdehyde; H_2_O_2_, hydrogen peroxidase; Phenol, total phenolic content; DPPH, antioxidant activity.

The correlational analysis provided a brief remarkable positive relationship among EL, all antioxidant enzymes activity, and proline, besides between proline and all antioxidant enzymes activity. In contrast, significant negative correlations were observed among all antioxidant enzymes activity, proline, and EL against RWC. It was found some notable positive relationships between MDA, EL, all antioxidant enzymes activity, and total soluble carbohydrates. Moreover, there were significant positive correlations between phenolic content and RWC as well as phenolic content and carotenoid, and eventually between DPPH, RWC, and carotenoid (Figure 2).

## 3. Discussion

Drought stress is one of the most important environmental stresses that has a negative effect on physiological and biochemical changes and finally on the growth and yield of plants. On the other hand, different genotypes and cultivars have different reactions in response to drought [3]. In an experiment on strawberry cultivars, the greatest yield was observed in slight drought stress and there was no significant difference between control and slight stress, although severe water deficit revealed the lowest yield [18]. Strawberry cultivars differed in their response to various water supplies due to different water requirements. This difference can be attributed to their biomass partitioning into vegetative/reproductive organs [19]. In other words, by considering the amount of water requirement in different strawberry cultivars, it can be achieved the highest yield for every special cultivar. Sensitive plants need a more precise irrigation schedule [20]. The results of this experiment are in agreement with Adiba et al. [21]. They reported severe drought stress causes obvious differences among pomegranate cultivars. 

The present study tends to describe the biochemical and physiological mechanisms behind the response of two strawberry cultivars to drought as a negative factor influencing their growth and yield. Plants exposed to drought decreased their RWC. Camarosa strawberry plants in our study displayed relatively higher RWC in slight and moderate drought stress. In general, tolerant plants respond to water deficit by stomatal closure and minimizing water loss. This RWC adjustment is associated with plant adaptation to water deficiency [22]. In another study, the reaction of three strawberry cultivars (Elsanta, Elkat, and Salut), different in terms of their response to water deficiency, was assayed. ‘Elsanta’ manifested a high rate of net photosynthesis as well as high water use efficiency (as an important ratio of photosynthesis rate to transpiration rate) under water deficit. Meanwhile, ‘Elsanta’ was considered to be the most drought-tolerant, which was reflected by both yield and growth characteristics [23]. Also in other studies, among 10 strawberry cultivars, Elvira has been regarded as the most drought tolerant due to its low transpiration rate and higher water use efficiency [24].

Both strawberry cultivars exposed to the drought stress conditions in our study indicated less total Chl and carotenoids. However, although Camarosa had an initially higher Chl content than Gaviota at normal growth conditions, Gaviota showed a lower decline in Chl under severe drought stress. For drought resistance, leaf Chl content is critical due to its direct effect on photosynthetic efficiency. This trait diminished during severe water stress and also in two other varieties of strawberries [25]. Increased accumulation of ROS in drought stress conditions favors the disruption of Chl molecules and eventually, the photosynthetic performance declined [26]. Water shortage negatively affects the intercellular CO_2_ levels which leads to the decrease of constituents in photosynthetic electron transport. As a result, ROS generation is enhanced which causes the degradation of the photosynthetic apparatus [27]. 

Camarosa displayed more active antioxidant enzymes and antioxidant capacity (DPPH) as compared to Gaviota in drought stress. It could be regarded as a response to drought stress-induced oxidative damage, while CAT, APX, POD, and SOD as defensive strategies play a crucial role in conserving plants against free oxygen species [28]. The maintenance of the proper function of the antioxidant system is essential to mitigate the damage of ROS accumulation in drought stress [29]. Under water deficit, antioxidant activity severely depends on plant species and cultivar and this activity is higher in tolerant cultivars [30]. The severity of damage of oxidative stress to the cell membrane is distinguished via H_2_O_2_ and MDA is often formed as a reliable estimator for lipid peroxidation. In another word, the indicator of lipid peroxidation is linked to the MDA measurement [31]. Oxidative stress resulting from drought stress led to enhancement of MDA and Camarosa elucidated less H_2_O_2_ comparing Gaviota cultivar whereas the level of MDA was higher in Camarosa conversely. In plant cells exposed to drought stress, abscisic acid (ABA) biosynthesis is elevated. Higher accumulation of ABA favor ROS production, causing oxidative stress in terms of enhanced EL due to lipid peroxidation in a membrane. Therefore, EL is measured as an estimator of the effect of drought stress on membrane permeability [32]. Both cultivars studied in our experiments showed enhanced EL, however, the EL was considerably enhanced especially in Gaviota. Drought stress increased oxidative markers and EL in Iranian and American pistachio rootstocks remarkably, and more tolerant cultivars showed a decrease in these parameters [33].

With increasing drought severity, leaf proline increased in both strawberry cultivars. The stimulated osmolytes biosynthesis is a part of the mechanism to maintain the osmotic balance in drought stress conditions [27]. Proline is considered an osmoprotectant that contributes to cell turgor maintenance during stress and as an amino acid that facilitates the synthesis of prospective proteins responding to drought stress [34]. Proline by contributing to decreasing lipid peroxidation and defending cell redox potential and diminishing ROS level causes to maintains membrane integrity [35]. The low activity of proline dehydrogenase plays a significant role in proline accumulation. The proline dehydrogenase activity (as a result of *PDH* gene expression) progressively decreased with the duration of exposure to stress [36]. In another experiment to compare drought tolerance between two strawberry cultivars, the Festival cultivar showed more proline content in strong drought stress as compared with Fortuna using in vitro techniques [37]. Soluble carbohydrates are another effective osmolytes that accumulate under drought stress. Camarosa accumulated more soluble carbohydrates in all drought stress levels compared to Gaviota suggesting its better adjustment to drought-related stress.

Undoubtedly, phenolic compounds can assist in the enhancement of plant tolerance through the protection of membrane lipids against peroxidation by inhibiting the initiation of oxidizing chain reactions [38]. Drought stress impacts the flavonols production pathway by decreasing the endogenous enzymes or declining the expression of genes related to polyphenols [39]. Both cultivars in our study reduced the total phenolics in accordance with an increased level of drought stress, and in the end Camarosa showed higher total phenolics content suggesting its better adaptation to drought.

## 4. Material and Methods

### 4.1. Plant Material and Treatment

The present study was carried out in 2021 using the American strawberry cultivars (Camarosa and Gaviota) under greenhouse conditions with a 24/16 ± 5 °C day/night temperature range, 70 ± 5% relative humidity range, and approximately 12.5 h local natural photoperiod with no artificial lightening (Maragheh, East Azarbaijan province, Iran; 37°30′ N, 46°12′ E, altitude 1477.7 m). Camarosa, as a short-day, early maturating cultivar, characterized by strength growth, big and hard fruit, and high yield, and Gaviota, cultivar with higher diseases resistance, and big and hard fruits with suitable favor, have been selected for this experiment [40]. Strawberry daughter plants were obtained from the commercial nursery in the Kurdistan province of Iran. Bare-root strawberry plants were cultivated (three-leaf stage) in plastic pots (5 kg) filled with field soil and irrigated for two weeks (every 3 days). Before applying drought stress, the plants were fed with Hoagland nutrient solution for better establishment. From late March, four irrigation regimes were established [100% FC (field capacity) (control), 75% FC (slight), 50% FC (moderate), and 25% FC (severe)]. FC was established following the method of Souza et al. [41] with the gravimetric procedure by daily weighing of the pots (including plants) and replacing the water lost during transpiration with a precision scale. These treatments continued until early May and at the end of the experiment (after the fruit set), plant samples (leaves) were harvested in each treatment to evaluate the physiological and biochemical parameters.

### 4.2. Photosynthetic Pigments, EL, and RWC

Leaf samples were extracted with 80% acetone and the absorbance was recorded by spectrophotometry (Carry 100, Richmond, VA, USA). Total Chl and carotenoids were measured according to Arnon [42]. In order to measure EL, washed leaf discs were placed in 20 mL of distilled water, shaken for 24 h, and EL1 was measured via EC-meter. The leaves were then located in the autoclave for 20 min (121 °C), cooled, and EL2 was measured accordingly. The EL was finally calculated using Equation (1) [43]:

EL(%) = (EL1/EL2) × 100(1)

According to Gucci et al. [44] method, the fresh weight (FW) of leaves was measured first and the leaves were then rehydrated for 20 h. Afterward, the leaf turgid weight (TW) was determined and the same leaves were dried at 80 °C to measure dry weight (DW) for 48 h. RWC was determined through Equation (2):

RWC = [(FW − DW)/(TW − DW)] × 100(2)

### 4.3. Antioxidant Enzymes Activity

In order to measure the enzyme activity, 0.5 g of the leaf samples were initially homogenized with 50 mM potassium phosphate (K-P) buffer (ice-cold). The homogenized extractions were then centrifuged and the supernatant was used to determine the enzyme activity.

The peroxidase (POD) (EC 1.11.1.7) activity was assayed in a 700 µL reaction mixture containing K-P buffer (25 mM, pH 7.0), H_2_O_2_ (10 mM), 0.05% guaiacol and 5 µL of the crude enzyme extract. The increasing tendency of the recorded absorbance was monitored at 470 nm for 1 min and the extinction coefficient of 26.6 mM^−1^ cm^−1^ [45]. The activity of catalase (CAT) (EC 1.11.1.6) was estimated by monitoring the decreasing trend in absorbance at 250 nm for 1 min. the extinction coefficient of 39.4 mM^−1^ cm^−1^ was applied to calculate CAT activity [46]. Superoxide dismutase (SOD) (EC 1.15.1.1) activity was measured following the protocol explained by Mostofa et al. [47]. The reaction mixture included 5 µL enzyme solution and 50 mM K-P buffer (pH 7.0), nitro-blue tetrazolium (2.24 mM), catalase xanthine (2.36 mM), and 0.1 unit of xanthine oxidase. To determine the activity of ascorbate peroxidase (APX) (EC 1.11.1.11) based on the method of Nakano and Asada [48], the change of absorbance at 290 nm during 1 min was recorded spectrophotometrically. The solution mixture contained 50 mM K-P buffer (pH 7.0), 0.5 mM ASA, 0.1 mM EDTA, 5 µL enzyme extract, and 0.1 mM H_2_O_2_. 

### 4.4. Oxidative Markers

H_2_O_2_ was determined according to the method of Alexieva et al. [49]. The reaction mixture included 0.5 mL leaf extract, trichloroacetic acid (TCA, 0.1%), K-P buffer (0.5 mL, 100 mM), and 2 mL KI (1 M) in double distilled water. After storing the reaction in darkness (1 h), absorbance was read at 390 nm. 

Malondialdehyde (MDA) levels were determined according to the procedure described by Hodges et al. [50]. Three mL of 0.5% thiobarbituric acid (TBA) and 2 mL of extraction solution were mixed severely at 95 °C as a constant temperature water bath for 30 min and then cooled on the ice to achieve room temperature. The concentration of MDA (C_MDA_) was calculated through Equation (3):

C_MDA_ (nmol·g^−1^ fresh weight) = 6.45 × (D_532_ − D_600_) − 0.56 × D_450_ (3)

where D_450_, D_532_, and D_600_ indicate thespectrophotometric absorbance at 450, 532, and 600 nm, respectively.

### 4.5. Osmolytes (Proline and Soluble Carbohydrates)

Initially, ninhydrin (1.25 g) was dissolved in 30 mL glacial acetic acid with 20 mL phosphoric acid, accompanied by warming. The reagent was then maintained at 4 °C (24 h). Plant material (0.5 g) was homogenized in 10 mL of aqueous sulfosalicylic acid. The resulting homogenate was filtered using Whatman filter paper and 2 mL of the obtained filtrate was added to 2 mL of glacial acetic acid with acid-ninhydrin (2 mL) at 100 °C (1 h) followed by the use of an ice-bath to stop the reaction. Vigorous vortexing was then performed for 15–20 s before warming (to reach room temperature) and recording the absorbance at 520 nm. The proline concentration was calculated using a calibration curve based on Equation (4) [51] as follows:

[(µg proline·mL^−1^ × mL toluene)/115.5 µg·µmole^−1^]/[(g sample)/5] = µmoles·g^−1^ of fresh weight material. (4)

Total soluble carbohydrates were determined according to Yemm and Willis [52]. Anthrone was dissolved in sulfuric acid (*v*/*v*, 45 mL). The reaction mixture consisted of 50 µL of the alcoholic extract and 950 µL deionized water (the final volume of 1000 µL). The anthrone solution (2000 µL) was then supplemented to the reaction while kept in an ice bath, followed by incubation at 100 °C for 3 min and cooling down to room temperature. Glucose was used as the standard to read the absorbance at 620 nm (as mg·g^−1^ of fresh weight).

### 4.6. Phenolic Content and Antioxidant Activity

Total phenolics content was determined according to a protocol described by Singleton and Rossi [53] using the Folin-Ciocalteu reagent. The reagent reacted with the diluted sample (0.5 mL) for 4 min and then a 2 mL solution of saturated sodium carbonate was added to the reaction mixture. Following storing at room temperature (2 h), the recording of absorbance was conducted at 760 nm and the results were expressed as mg gallic acid in 100 g FW of plant material based on a gallic acid standard. The measurement of antioxidant activity was adapted by Brand-Williams et al. [54] with the free radical (DPPH). Antioxidant solution (0.1 mL) in methanol was added to 3.9 mL of DPPH solution. The reducing trend at 515 nm was monitored every 1 min interval to reach a stable record. The reduction was measured as the Equation (5):

Abs _515 nm_= 12.509 × (C_DPPH_) − 2.58 × 10^−3^ (5)

where C_DPPH_ is the initial DPPH concentration obtained from a calibration curve.

### 4.7. Statistical Analysis

The factorial experiment was carried out as a completely randomized design (CRD) with three replicates. A two-way analysis of variance (ANOVA) was performed using the General Linear Model procedure to calculate the effects of drought stress (four irrigation regimes) and cultivars (two cultivars). ANOVA was performed with SAS 9.1 (SAS Institute Inc., Cary, NC, USA). Duncan’s multiple range test was applied in SAS to compare the significance of the treatment’s mean values at *ρ* < 0.05.

## 5. Conclusions

Arid and semi-arid conditions overcome in most parts of the world including Iran and drought as a major abiotic environmental obstacle has been restricting plant production. Therefore, cultivars that can withstand drought are in high demand. Strawberry as the most important small fruit crop is seriously affected by this abiotic stress. It is clear that the drought tolerance variation found between Camarosa and Gaviota in this investigation could be associated with antioxidant activity. In general, it might be concluded that the Camarosa due to enhanced activity of antioxidant enzymes (CAT, SOD, and APX) and increased phenolic content as non-enzymatic antioxidants, respectively, as well as soluble sugars as effective osmoprotectants, is more adapted to water deficit. On the other hand, increased levels of H_2_O_2_ and higher EL accompanied by fewer enzymatic antioxidants in response to drought stress confirms less tolerance of the Gaviota cultivar to water deficit. Whether efforts to maximize drought tolerance by improving water use efficiency in these strawberry cultivars need to be, however, evaluated in further experiments.

## Figures and Tables

**Table 1:** Effects of different drought stress levels on photosynthetic pigments [total chlorophyll (Total Chl) and carotenoid (CAR) content], electrolyte leakage (EL), and relative water content (RWC) in Camarosa and Gaviota strawberry cultivars.

Cultivar	Drought Stress Level	Total Chl	CAR	EL (%)	RWC (%)
		(mg g^−1^ FW)			
Camarosa	Control ^1^	3.69 ± 0.08 a	1.34 ± 0.16 ab	17.32 ± 0.93 f	87.31 ± 1.35 a
	Slight ^2^	3.21 ± 0.07 b	1.24 ± 0.24 bc	21.52 ± 0.61 e	78.38 ± 2.23 b
	Moderate ^3^	2.35 ± 0.07 e	1.13 ± 0.26 cd	33.52 ± 0.66 c	66.52 ± 1.04 d
	Severe ^4^	1.07 ± 0.11 f	0.83 ± 0.21 e	39.26 ± 0.71 b	59.24 ± 3.21 ef
Gaviota	Control ^1^	2.85 ± 0.09 c	1.44 ± 0.13 a	14.60 ± 1.19 g	83.55 ± 1.75 a
	Slight ^2^	2.61 ± 0.11 d	1.35 ± 0.25 ab	22.60 ± 1.06 e	74.26 ± 2.72 c
	Moderate ^3^	2.49 ± 0.10 de	1.02 ± 0.08 d	28.57 ± 0.70 d	61.66 ± 3.68 e
	Severe ^4^	2.37 ± 0.10 e	0.68 ± 0.18 e	41.26 ± 0.85 a	55.65 ± 3.64 f
	**df**	**Mean Square**			
Cultivar	1	0.0024 ^ns^	0.0008 ^ns^	7.878 ^ns^	82.770 **
Drought	3	2.831 **	0.486 **	675.909 **	975.481 **
Cultivar × Drought	3	1.294 **	0.028 *	15.869 **	1.993 *

^1^ 100% FC (field capacity), ^2^ 75% FC, ^3^ 50% FC, ^4^ 25% FC. ns, *, ** non-significant or significant at *p* < 0.05 and 0.01, respectively. Values are means ± SE (n = 3). Different letters within the same column indicate significant differences at *p* < 0.05 among the treatments, using Duncan’s multiple range test.

**Table 2:** Effects of different drought stress levels on antioxidant enzyme activity (peroxidase (POD), catalase (CAT), superoxide dismutase (SOD), and ascorbate peroxidase (APX)) in two strawberry cultivars.

Cultivar	Drought Stress Level	POD	CAT	SOD	APX
		(μmol min^−1^ mg^−1^ Protein)	(μmol min^−1^ mg^−1^ Protein)	(U min^−1^ mg^−1^ Protein)	(μmol min^−1^ mg^−1^ Protein)
Camarosa	Control ^1^	4.04 ± 0.15 g	0.05 ± 0.01 de	14.79 ± 0.93 e	1.39 ± 0.10 e
	Slight ^2^	4.93 ± 0.30 ef	0.07 ± 0.06 d	15.56 ± 0.89 de	1.53 ± 0.25 de
	Moderate ^3^	7.79 ± 0.19 d	0.11 ± 0.01 b	17.81 ± 0.84 c	1.74 ± 0.11 cd
	Severe ^4^	9.59 ± 0.21 b	0.16 ± 0.01 a	21.76 ± 1.18 a	3.12 ± 0.09 a
Gaviota	Control ^1^	4.65 ± 0.27 f	0.03 ± 0.01 e	14.36 ± 0.10 e	1.45 ± 0.18 e
	Slight ^2^	5.16 ± 0.19 e	0.04 ± 0.01 e	15.34 ± 1.11 d e	1.72 ± 0.23 cd
	Moderate ^3^	8.48 ± 0.43 c	0.09 ± 0.01 c	16.35 ± 0.95 d	1.87 ± 0.12 c
	Severe ^4^	10.28 ± 0.37 a	0.12 ± 0.03 b	19.83 ± 0.62 b	2.80 ± 0.14 b
	**df**	**Mean Square**			
Cultivar	1	1.859 **	0.003 **	6.101 *	0.008 ^ns^
Drought	3	41.372 **	0.014 **	45.414 **	0.032 **
Cultivar × Drought	3	0.072 *	0.00003 *	1.004 *	0.054 *

^1^ 100% FC, ^2^ 75% FC, ^3^ 50% FC, and ^4^ 25% FC. ns, *, ** non-significant or significant at *p* < 0.05 and 0.01, respectively. Values are means ± SE (n = 3). Different letters within the same column indicate significant differences at *p* < 0.05 among the treatments, using Duncan’s multiple range test.

**Table 3:** Effects of different drought stress levels on oxidative markers [hydrogen peroxide (H_2_O_2_) and malondialdehyde (MDA)] and osmolytes (proline and total soluble carbohydrates) in two strawberry cultivars.

Cultivar	Drought Stress Level	H_2_O_2_	MDA	Proline	Total Soluble Carbohydrates
		(nmol g^−1^ FW)		(µmol g^−1^ FW)	(mg g^−1^ FW)
Camarosa	Control ^1^	4.85 ± 0.19 d	4.23 ± 0.16 c	1.18 ± 0.11 d	16.12 ± 0.15 d
	Slight ^2^	5.02 ± 0.29 d	4.37 ± 0.11 c	1.26 ± 0.14 cd	16.60 ± 0.11 c
	Moderate ^3^	5.54 ± 0.17 c	5.04 ± 0.08 b	1.35 ± 0.08 cd	17.18 ± 0.26 b
	Severe ^4^	6.58 ± 0.11 b	5.43 ± 0.12 a	2.02 ± 0.17 b	23.18 ± 0.17 a
Gaviota	Control ^1^	3.89 ± 0.12 f	3.09 ± 0.13 e	1.24 ± 0.13 cd	13.50 ± 0.33 f
	Slight ^2^	4.28 ± 0.24 e	3.53 ± 0.10 d	1.42 ± 0.17 c	13.94 ± 0.27 e
	Moderate ^3^	6.37 ± 0.10 b	4.14 ± 0.19 c	1.85 ± 0.18 b	15.85 ± 0.21 d
	Severe ^4^	7.56 ± 0.11 a	4.93 ± 0.15 b	2.82 ± 0.06 a	16.72 ± 0.20 c
	**df**	**Mean Square**			
Cultivar	1	0.007 ^ns^	4.293 **	0.870 **	63.342 **
Drought	3	9.419 **	2.748 **	1.774 **	98.916 **
Cultivar × Drought	3	1.589 **	0.104 **	0.173 **	21.279 **

^1^ 100% FC, ^2^ 75% FC, ^3^ 50% FC, ^4^ 25% FC. ns, *, ** non-significant or significant at *p* < 0.05 and 0.01, respectively. Values are means ± SE (n = 3). Different letters within the same column indicate significant differences at *p* < 0.05 among the treatments, using Duncan’s multiple range test.

## Data Availability

The datasets generated and analyzed during the current study are available from the corresponding author upon reasonable request.
